# Identification of genetic loci associated with forage quality in response to water deficit in autotetraploid alfalfa (*Medicago sativa* L.)

**DOI:** 10.1186/s12870-020-02520-2

**Published:** 2020-07-01

**Authors:** Sen Lin, Cesar Augusto Medina, Bill Boge, Jinguo Hu, Steven Fransen, Steven Norberg, Long-Xi Yu

**Affiliations:** 1 United States Department of Agriculture-Agricultural Research Service, Plant Germplasm Introduction and Testing Research, 24106 N Bunn Road, Prosser, WA 99350 USA; 2 Irrigated Agriculture Extension and Research Center, Washington State University, 24106 N Bunn Road, Prosser, Washington USA; 3 Washington State University Franklin County Extension Office, 404 West Clark Street, Pasco, Washington USA

**Keywords:** NIRS, Drought, GWAS, Genetic loci, Phenotypic variation, Genetic diversity

## Abstract

**Background:**

Alfalfa has been cultivated in many regions around the world as an important forage crop due to its nutritive value to livestock and ability to adapt to various environments. However, the genetic basis by which plasticity of quality-relevant traits influence alfalfa adaption to different water conditions remain largely unknown.

**Results:**

In the present study, 198 accessions of alfalfa of the core collection for drought tolerance were evaluated for 26 forage quality traits in a field trial under an imposed deficit irrigation gradient. Regression analysis between quality traits and water stress revealed that values of fiber-related traits were negatively correlated with values of energy-related traits as water deficit increased. More than one hundred significant markers associated with forage quality under different water treatments were identified using genome-wide association studies with genotyping by sequencing. Among them, 131 markers associated with multiple traits in all the water deficit treatments. Most of the associated markers were dependent to the levels of water deficit, suggesting genetic controls for forage quality traits were dependent to the stress treatment. Twenty-four loci associated with forage quality were annotated to functional genes that may play roles in cell development or in response to water stress.

**Conclusions:**

This study addressed the genetic base of phenotypic variation of forage quality traits under water deficit. The SNP markers identified in this study will be useful in marker-assisted selection for the genetic improvement of alfalfa with enhanced drought tolerance while maintaining forage quality.

## Background

Alfalfa, “Queen of the Forages”, is the most productive and highest quality forage crop. Alfalfa quality has been determined by many factors, including protein, fiber and lignin contents, relative feed value, total digestible nutrients, and other physical and chemical factors. Alfalfa quality is directly influenced by its feeding value from animal performance. Fiber contents such as acid detergent fiber (ADF) and neutral detergent fiber (NDF) are important factors affecting the forage quality. Alfalfa forage contains 35–55% NDF, which contributes ~ 20–30% of the digestible energy value of alfalfa, the rest coming from non-fiber components. Relative feed value (RFV) is a tool that indexes alfalfa quality primarily based on its NDF content. The RFV index estimates digestible dry matter (DDM) of the alfalfa from ADF, and calculates the dry matter intake (DMI) potential from NDF. However, RFV has a significant shortcoming because it does not take into account how variations in NDF digestibility affect the energy content or intake potential of alfalfa. Even when harvested at an immature stage, the digestibility of alfalfa fiber can be very different [1]. In 2004, scientists at the University of Wisconsin designed another index, relative forage quality (RFQ) for estimating forage quality. The RFQ uses fiber digestibility and the total digestible nutrients of the forage to estimate intake [2]. The RFQ is an improvement over RFV as it better reflects the performance on animal fed. The RFQ emphasizes fiber digestibility while RFV uses digestible dry matter intake. The RFV continues to be widely used as an index to assess quality, compare forage varieties, and price forages. However, differences in the digestibility of the fiber fraction can result in a difference in animal performance when forages with a similar RFV index are fed.

Alfalfa forage quality and yield is affected by environmental factors, such as soil salinity and water supply. Alfalfa yield significantly declines when irrigation was not adequate [3]. Irrigation in an arid climate can affect nutritive value of alfalfa hay. Drought and high salinity are major factors that affect plant growth in the arid and semi-arid regions. Plants cope with these challenges by stress-avoidance or stress-tolerance. Stress-tolerant plants have evolved certain adaptive mechanisms within phenotypic plasticity to achieve different degrees of tolerance, whereas stress avoidance is the ability of plants to minimize the adverse effect.

The extent of phenotypic plasticity is primarily determined by genetic changes. Plants evolved mechanisms that facilitate adaptation to environmental changes under selective pressure [4]. It is unclear whether plant phenotypic plasticity is controlled by specific genes or a result of epistatic interaction during the selection of individual traits. However, genetic diversity and heterozygosity enhance adaptability to variable environments. It is important to identify plant functional traits and plasticity which will help plants adapt to global climate change [5].

Identification of molecular markers is the first step in marker-assisted breeding for genetic improvement. Single nucleotide polymorphism (SNP) is a type of markers that widely exist throughout the genome. One of the high-throughput and highly efficient approaches to discover SNPs is genotyping by sequencing (GBS), which was primarily used for phylogenesis and genome-wide association studies (GWAS) in maize [6]. It based on the high-throughput next generation sequencing [7]. Compared to the commercial SNP arrays, GBS has more advantages as its low cost, time saving and easy automation [8]. GWAS requires a large number of markers for mapping complex traits at the whole genome level. Over 15,000 SNPs were used to identify significant markers associated with cell wall biosynthesis and biomass yield in *M. sativa* [9]. In alfalfa, Li et al. [10] used GBS markers to construct a high-density linkage map representing high synteny between linkages of *M. sativa* and its wild relative, *M. truncatula*.

Unlike traditional genetic mapping, GWAS use thousands of SNPs throughout the genome to identify quantitative trait loci (QTL) associated with traits of interest using linkage disequilibrium [11]. Several factors can potentially influence GWAS power to identify significant associations, such as phenotypic variation, individual number, allele frequency, and population structure [12]. Mixed models have been used to correct the population structure and reduce the false positives of marker-trait association [13]. GWAS has been successfully used for mapping agronomic traits in some major crops, such as rice, maize, wheat, sorghum and soybean [14].

Alfalfa is an autotetraploid species (2n = 4X = 32) and alfalfa plants are highly heterozygous. It is a considerable challenge to develop markers with allele dosage in such a complex genome [15]. Since alfalfa cultivars are genetically broad-based synthetic populations, they provide an ideal system in which GBS, GWAS, and genomic selection (GS) can be applied. GWAS have been used for mapping quantitative trait loci associated with biomass yield, biotic and abiotic stresses in alfalfa [16]. Genetic markers associated with forage quality were identified by GWAS [17]. In addition, nearly 10,000 SNP markers were used for GS modeling, and further showed that GS increased genetic gain of biomass yield in alfalfa [18].

In the present study, we evaluated 26 forage quality traits in a panel of 198 alfalfa accessions of the core collection for drought tolerance obtained from the USDA-ARS Western Regional Plant Introduction Station. Majority of the accessions were collected in 1980s, in Canada and Northern US and dryland regions of other countries. The plants were evaluated in the field under three irrigation regimes: well-watered, mild and severe water deficits. To investigate the genetic base of the forage quality and its interaction with water stress treatments, we applied high-throughput genome sequencing GBS followed by GWAS, an integrated framework merged a QTL mapping approach to investigate genomic architecture of phenotypic plasticity of alfalfa quality traits under a gradient of water deficits. The ultimate goal is to identify genetic markers associated with forage quality traits in alfalfa under a deficit irrigation gradient, and use the closely linked markers for marker-assisted selection in breeding for high quality alfalfa varieties based on genetic potential and to reduce the confounding of environmental conditions with traditional breeding methods.

## Results

### Phenotypic variations of forage quality traits

**Fig. 1:**
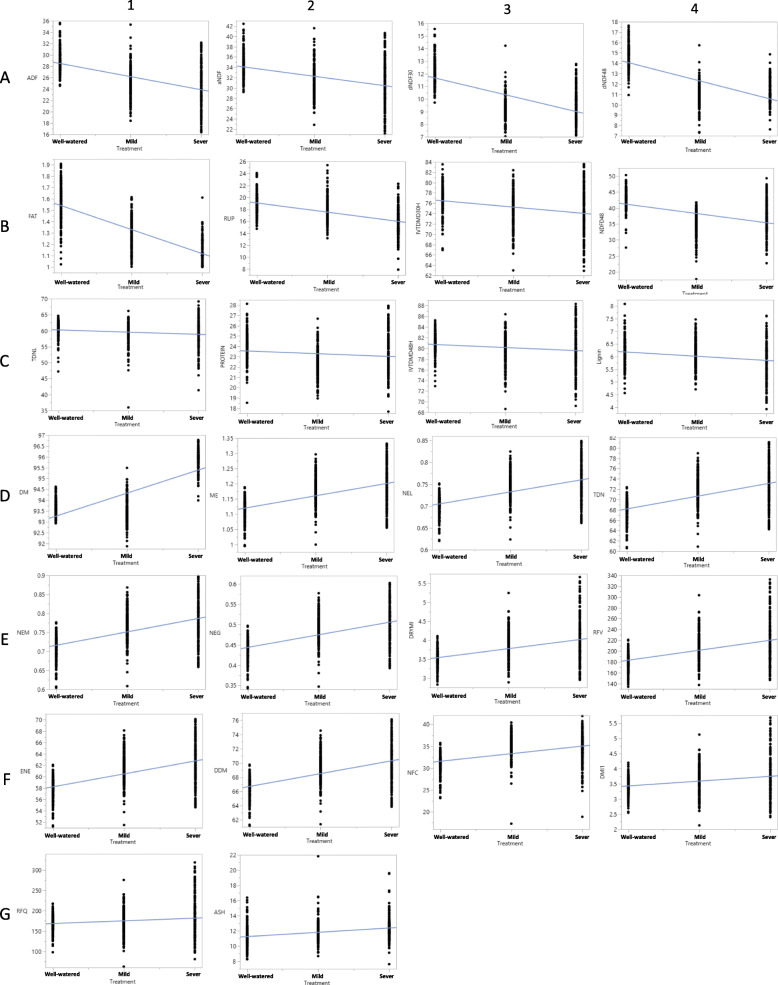
Regression plots for 26 forage quality traits showing phenotypic variations under well-watered, mild and severe water deficits (X-axis, from left to right). Phenotypic values of each trait are presented by Y-axis

The analysis of variance (ANOVA) for 26 quality traits was carried out among the panel of germplasm and the result is presented in Table 1. The sum of squares varied from 0.29 in net energy of lactation (NEL) to 130,770.31 in RFV. The differences of most of the traits are statistically significant with the probability < 0.0001. Regression analyses of phenotypic variations in different water conditions showed significant effects of water deficit on forage quality (Fig. 1). The values of fiber-related traits, including ADF, aNDF, dNDF30, dNDF48 decreased as water deficit applied (Fig. 1a 1–4). Water deficit also decreased the contents of fat, RUP, IVDDM30 and NDFD48 (Fig. 1b 1–4), and slightly decreased TDNL, protein, IVDDM48 and lignin (Fig. 1c 1–4). Whereas the values for energy-related traits including: ME, NEL, TDN, NEM, NEG, RFV, ENE, DDM, and NFC increased by water deficit (Fig. 1d, e, and f). Water deficit slightly increased RFQ and ash (Fig. 1g 1–2).

**Table 1:** Analysis of variances of forage quality traits in the panel of 198 alfalfa accessions

	Analysis of Variance				Parameter Estimates		
Variable	Sum of Squares	F Ratio	Prob > F	Estimate	Std Error	t Ratio	Prob>|t|
DM	437.53	711.77	<.0001	92.19	0.09	1076.20	<.0001
CP	26.72	9.45	0.0022	23.81	0.18	129.60	<.0001
ADF	2070.37	231.21	<.0001	30.82	0.33	94.25	<.0001
aNDF	1276.96	104.09	<.0001	35.90	0.38	93.80	<.0001
IVTDMD48	125.19	13.11	0.0003	81.29	0.34	240.76	<.0001
dNDF48	1176.80	548.38	<.0001	15.80	0.16	98.71	<.0001
IVTDMD30	577.79	45.29	<.0001	77.70	0.39	199.10	<.0001
dNDF30	681.32	386.22	<.0001	12.99	0.15	89.54	<.0001
ASH	121.61	58.16	<.0001	10.69	0.16	67.68	<.0001
FAT	17.27	920.86	<.0001	1.75	0.01	117.13	<.0001
Lignin	10.91	29.56	<.0001	6.36	0.07	95.74	<.0001
RUP	911.85	220.29	<.0001	20.61	0.22	92.70	<.0001
NEL	0.29	231.21	<.0001	0.68	0.00	174.08	<.0001
TDN	2364.05	231.21	<.0001	65.72	0.35	188.10	<.0001
ENE	2000.33	231.21	<.0001	55.94	0.32	174.08	<.0001
ME	0.64	231.21	<.0001	1.08	0.01	188.10	<.0001
NEM	0.48	230.93	<.0001	0.68	0.00	136.66	<.0001
NEG	0.37	230.73	<.0001	0.41	0.00	93.76	<.0001
DDM	1256.39	231.21	<.0001	64.90	0.25	254.80	<.0001
DMI	22.16	114.24	<.0001	3.30	0.05	68.52	<.0001
NDFD48	3279.66	153.65	<.0001	44.12	0.50	87.40	<.0001
RFV	130,770.31	138.82	<.0001	164.71	3.35	49.12	<.0001
NFC	1158.15	121.12	<.0001	29.84	0.34	88.33	<.0001
TDNL	187.28	13.66	0.0002	60.95	0.40	150.66	<.0001
DMI1	9.74	36.29	<.0001	3.27	0.06	57.82	<.0001
RFQ	17,203.35	14.21	0.0002	161.78	3.80	42.56	<.0001

**Fig. 2:**
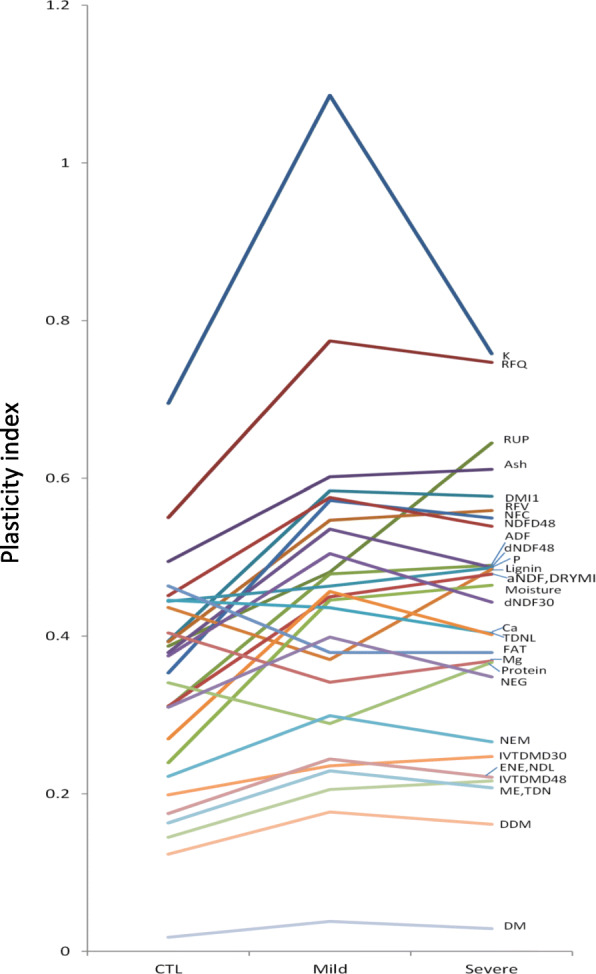
Phenotypic plasticity index of 26 alfalfa quality traits in response to well-watered (CTL), mild water deficit (Mild) and severe water deficit (Severe) conditions

Phenotypic plasticity was estimated by calculating the plasticity index (PI) for each trait in the given accession under well-watered and water deficit conditions as described in the section of Methods. Overall, higher PIs were found in water deficit conditions compared to well-watered control except lignin, fat and protein (Fig. 2). Within the water stress treatments, higher PIs were found in the mild stress than those in the severe stress for most of the quality traits. Among them, the highest PIs were found in RFQ with 0.55, 0.77 and 0.75 for the control, mild and severe drought conditions, respectively. The lowest PIs appeared in DMM with 0.12, 0.18 and 0.16 for control, mild and severe drought, respectively. The rest of the traits showed higher PIs in severe drought compared to the mild treatment. The PI values were very similar between DMI and aNDF, ENE and NEL, and TDN and ME.

**Fig. 3:**
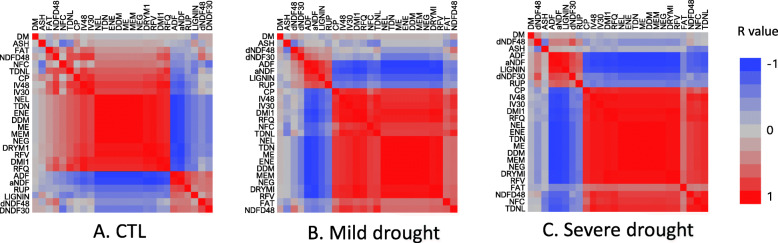
Correlation coefficient among 26 quality traits under well-watered control (CTL), mild water deficit (Mild) and severe water deficit (Severe) in alfalfa

The correlation coefficients (r) between fiber-related traits and energy-related traits were decreased as drought increased. Among them ADF, aNDF, RUP, lignin dNDF30 and dNDF48 were negatively correlated with other quality traits and the correlations were increased as drought increased (Fig. 3 a, b and c, blue panels). Positive correlations were found between energy-related traits (Fig. 3b and c, red panels on the bottom right). The correlations were increased as drought increased. Highest r values were found between these traits when the plants were under severe drought (Fig. 3c). However, no significant change was found between fat and any other traits by drought treatments (Fig. 3b and c).

### Cluster analysis of germplasm using forage quality traits

**Fig. 4:**
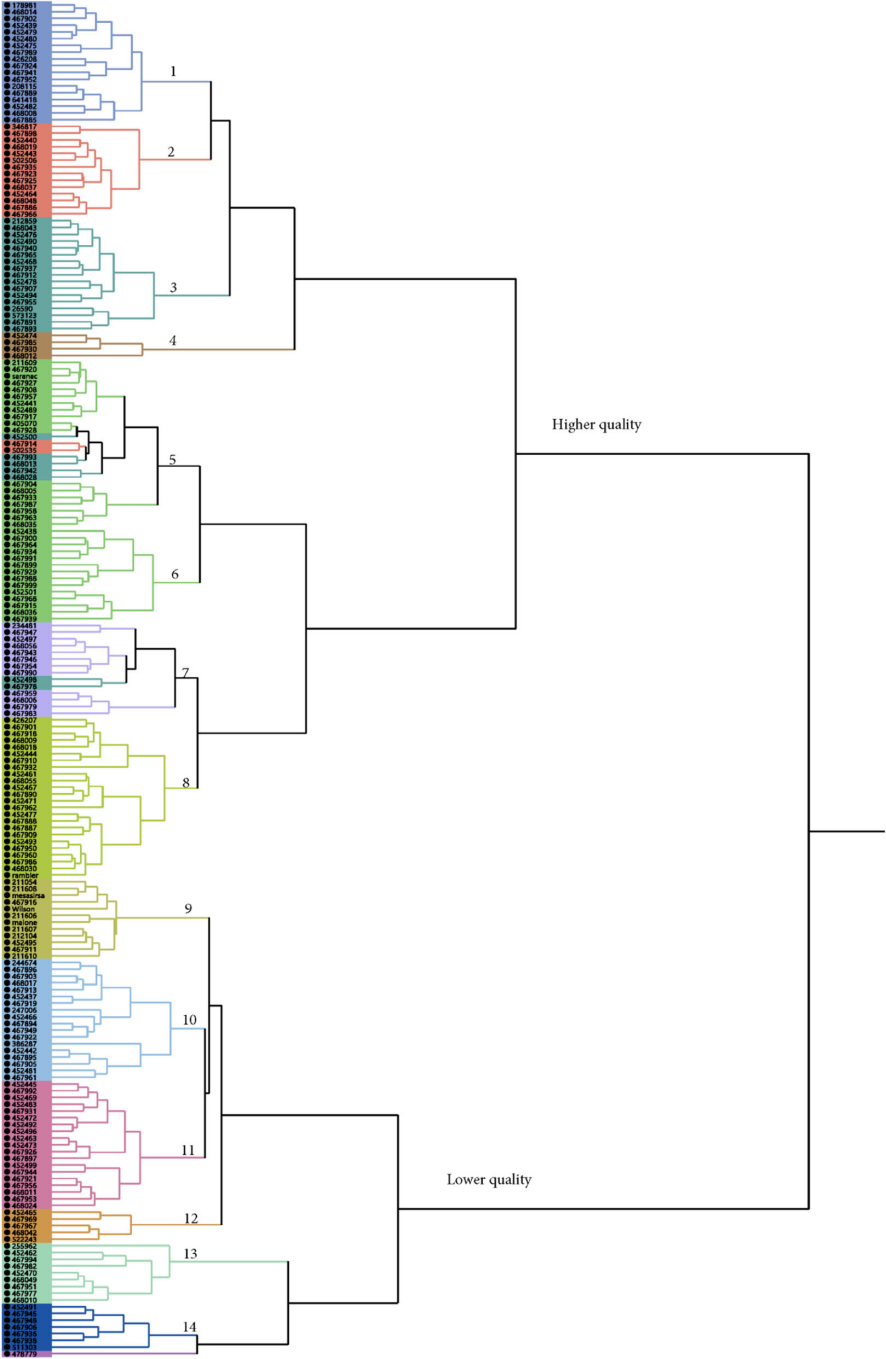
A hierarchical cluster obtained using farthest neighbor method with phenotypic values of all quality traits evaluated in the present study. Accessions (PIs) were clustered into 2 clusters (High and low quality clusters) and 14 subclusters. The high quality cluster contains 8 subclusters with relatively higher quality. The low quality cluster contains 6 subclusters with relatively lower quality. Two checks, “Saranac” and “Rambler”, susceptible to salt/drought stress were clustered into the high quality cluster (Subclusters 5 and 8, respectively), and three drought/salt resistance checks, “Mesa Sirsa”, “Wilson” and “Malone” were clustered into the low quality cluster (Subcluster 9)

The mean values of 26 forage quality traits were used for cluster analysis. Two large clusters and 14 subclusters were classified as showing in Fig. 4. The first large cluster contained 8 subclusters. Most of germplasm in this cluster were collected from cultivars from US and Canada and their quality traits such as crude protein and RFV were relatively higher (Table S1), so we named it as the higher forage quality cluster (Fig. 4, top cluster). Two checks, Rambler and Saranac, susceptible to salt/drought are in this cluster (Fig. 4, subclusters 5 and 8). The bottom cluster was furtherly classified into 6 subclusters containing germplasm collected worldwide, including old cultivars and landraces with relatively lower forage quality (Fig. 4, bottom cluster). Three salt/drought resistance checks, Malone, Mesa Sirsa and Wilson are in this cluster (Fig. 4 subcluster 9). There was a trend that alfalfa germplasm with resistance to salt/drought had lower forage quality, while higher quality was found in the susceptible alfalfa germplasm.

### Genome-wide association for forage quality

**Fig. 5:**
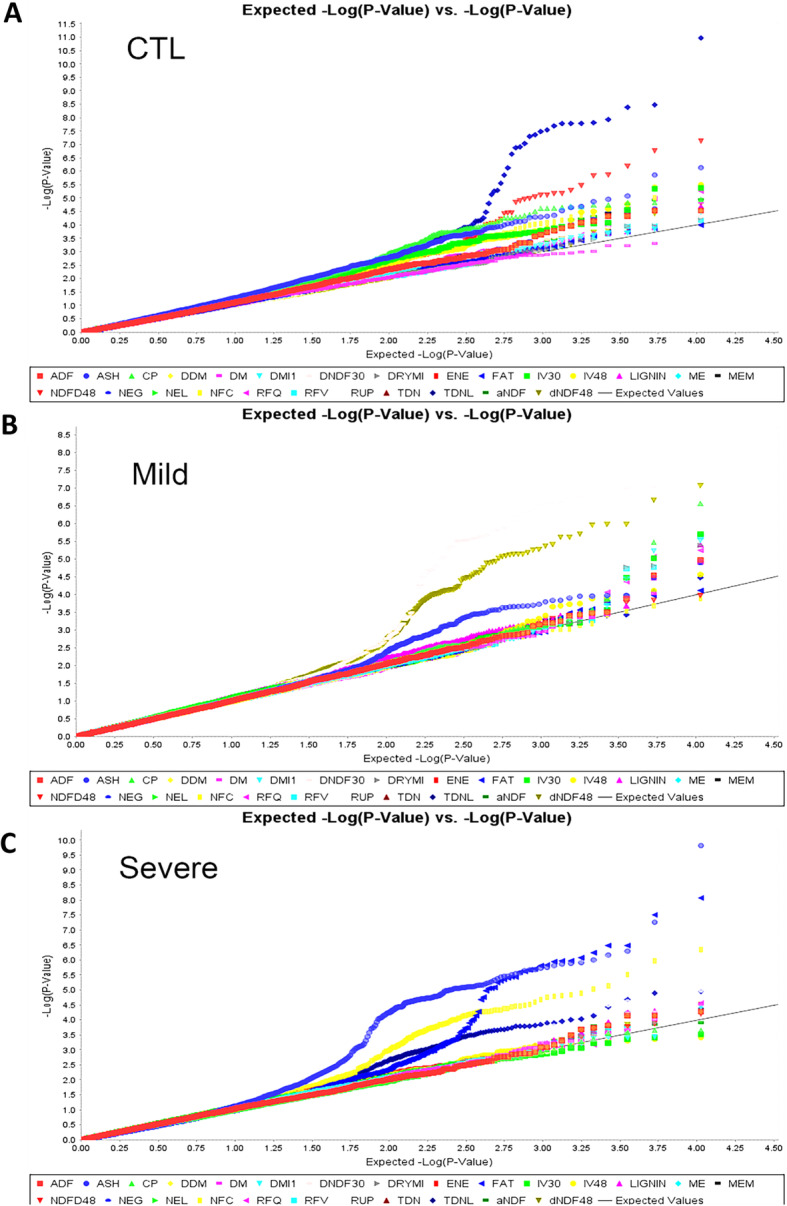
Quantile-quantile plots of marker-trait association from GWAS for forage quality traits under well-watered (**a**), mild (**b**) and severe (**c**) water deficits in the alfalfa association panel. The expected (solid lines) against observed (dot lines) -log10 p-values are presented on X-axis and Y-axis, respectively. Each color curve represents a quality trait as showing at the bottom of the figures

The combinations of the filtered 10,327 GBS markers and phenotypic data of 26 quality traits were analyzed by GWAS using a mixed linear model. The profile of marker-trait association for well-watered control (A), mild (B) and severe (C) water deficits were illustrated in quantile-quantile plots (QQ) (Fig. 5). As illustrated in Fig. 5, a consistent difference between expected (X-axis) and observed (Y-axis) *p*-value across the whole genome was implied by deviation from the X = Y. The significances of marker-trait association were presented in negative log *P*-values on the Y-axis. Only a small number of true associations were shown among majority of unassociated SNPs. Overall, lower significance was obtained for all traits in the control except ash (Fig. 5a). Most significant marker-trait association were found in quality traits under the mild stress (Fig. 5b). The level of significances was lower by the severe stress compared to that of the mild stress (Fig. 5c).

**Fig. 6:**
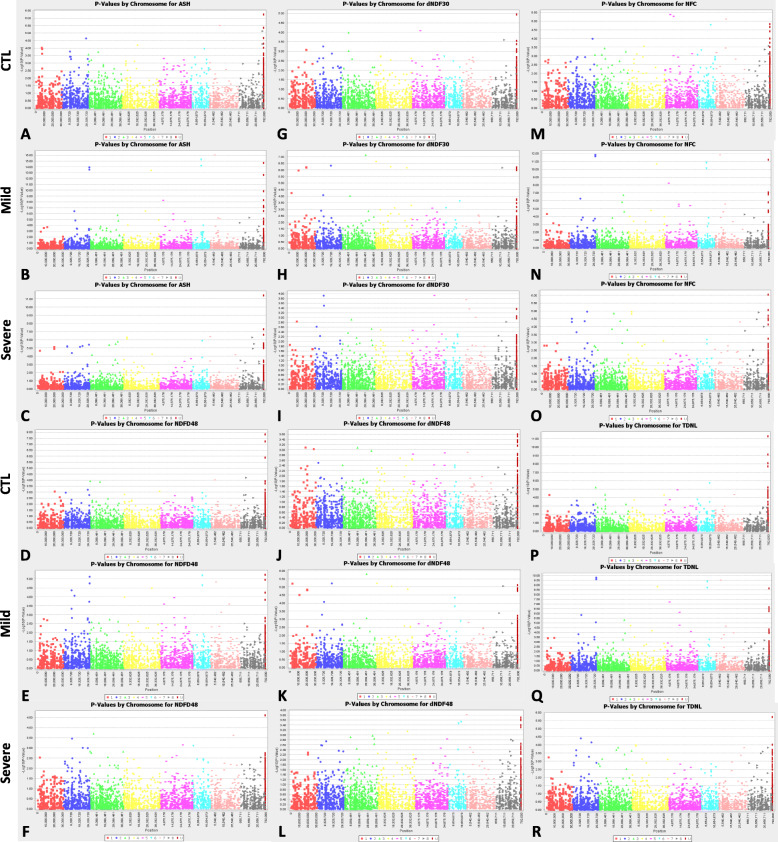
Manhattan plots of marker-trait association of six most significant quality traits under well-watered control (CTL), mild water deficit (Mild) and severe water deficit (Severe). The X-axis presents chromosome positions of loci based on the reference genome of *M. truncatula* (Mt4.0, v1). The Y-axis shows negative log (P-values) of marker-trait association. Chromosome numbers were assigned and illustrated at the bottom of the figures

Of 26 traits analyzed, most significant marker-trait associations were found in ash, NDFD48, dNDF30, dNDF48, NFC and TDNL. (Fig. 6 b, e, h, k, n and q). Similar association profiles were found in the severe stress, but the marker’s significances were lower than those under the mild stress (Fig. 6 c, f, i, l, o and r). Whereas, no or less significances were shown for the same markers under control condition (Fig. 6 a, d, g, j, m and p). Among markers identified, the highest significant markers were associated with ash and they were located on chromosomes 2, 6, 7 and unknown chromosome (U) (Fig. 6b). Significant markers associated with NDFD48 were also identified on same chromosomes (Fig. 6e) under mild stress but not in control and severe stress (Fig. 6 d and f). Significant markers were associated with dNDF30 and dNDF48 and they were located on chromosomes 1, 2, 3 and 8 (Fig. 6 h and k). Markers associated with NFC and TDNL were found in mild drought and they were located on chromosomes 2, 6 and 7 (Fig. 6, n, q).

### Common markers identified among multiple quality traits by different drought treatments

**Fig. 7:**
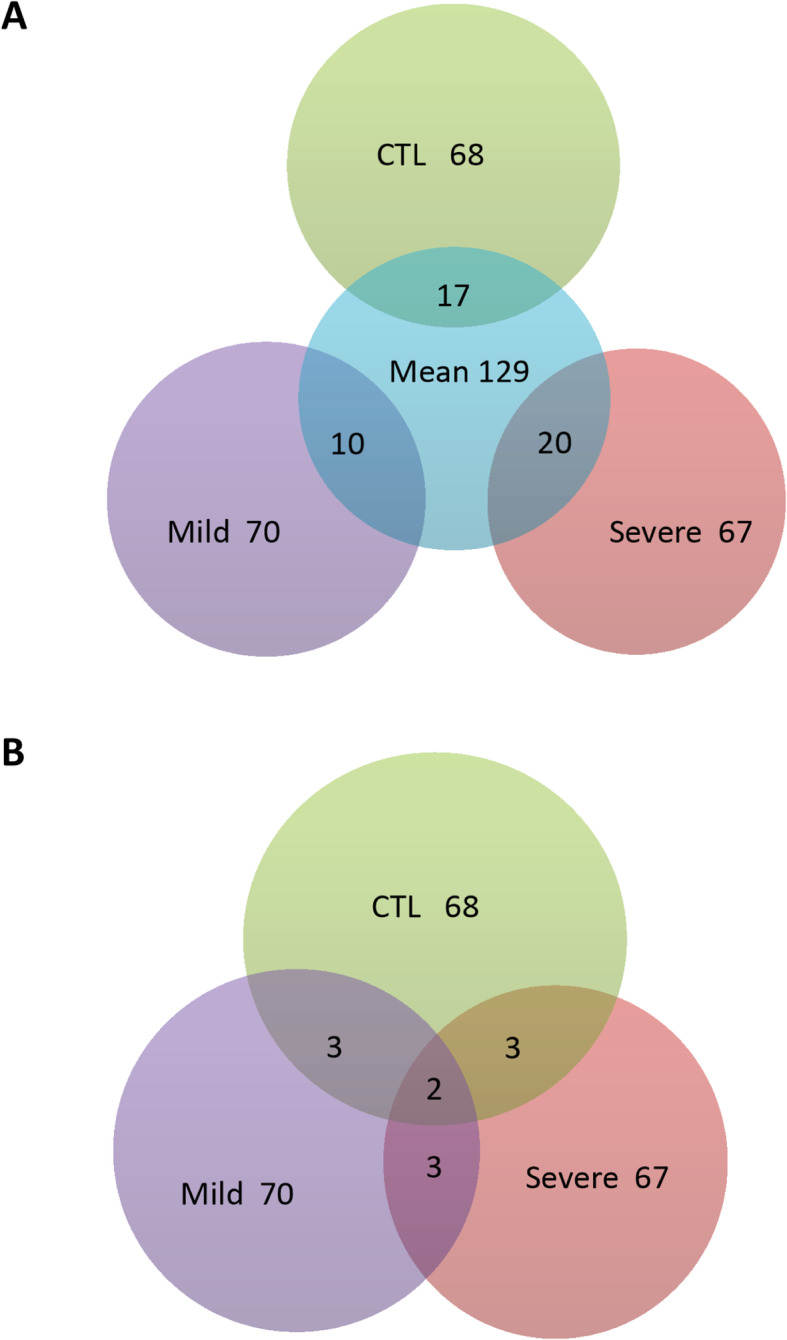
A Vann chart of significant loci associated with forage quality resulting from GWAS for quality traits in alfalfa under well-watered (CTL), mild and severe water deficits compared with mean (**a**) and without mean (**b**). The numbers of significant loci identified under each treatment were compared with those of mean values of all treatments, showing the numbers of common (overlapped) and specific loci for different treatments

Despite different loci identified among quality traits, common markers were found among multiple traits (Table 2). Marker S1_110050725 on chromosome 4 identified in CTL for ADF was also significantly associated with other 10 traits including DDM, ENE, IVDMD30, IVDMD48, ME, NEG, NEL NEM, Protein and TDN (Table 2, top row). Similarly, markers S1_305729816 for DMI1 in CTL was also associated with 6 other traits: IVDMD30, IVDMD48, NDFD48, RFQ, TNDL and crude protein contents. Marker S1_231443201 identified in ADF shared its association with DDM, ENE, ME, NEG, NEL, NEM, TDN and TDNL. Four markers (S1_197238737–90) at the same locus on chromosome 6 and unknown marker S1_292679040 identified for ash in the mild stress were also associated with 6 other traits. Markers S1_351118210 and S1_276968305 identified in CTL for IVDMD48 and ash, respectively, were significantly associated with 5 other traits. Marker S1_174013573 identified for DMI1 were also associated with DMI, fat, RFQ and RFV in the severe stress. Eight markers associated with ash also associated with NDFD48, NFC and TDNL (Table 2). Additionally, nine, eighteen and fifty-five markers identified in four, three and two traits, respectively. The remaining markers were associated with one trait (Table 2, bottom part). Interestingly, most of the high significant markers with lower *p* value and higher R^2^ are among of common markers, suggesting these markers may have major effects on forage quality under drought. Among those, ten markers were associated with three traits (NDFD48, NFC and TDN). The *p* values of these markers ranged from 4.01E-08 (S1_21394491) to 5.79E-16 (S1_197238737) and the marker’s R^2^ ranged from 0.22 to 0.38, respectively (Table 2). To oversee the genetic architecture of the population under different treatments, we compared markers significantly associated with CTL, mild and severe drought treatments with those identified markers using mean values of all treatments. Among 68 markers identified in the control, 17 were also identified in the mean (Fig. 7a). Of 70 significant markers identified in the mild stress, only 10 were also identified in the mean (Fig. 7a). Among 67 markers identified in severe drought, 20 were also found in the mean (Fig. 7a). We have also compared the common markers identified among the three treatments directly. There were 3 common markers between each pair of treatments (Fig. 7b). Only 2 markers were found in all three treatments (Fig. 7b).

**Table 2:** Significant markers associated with forage quality traits under well-watered control (CTL), mild water deficit (Mild) and severe water deficit (Severe) in the panel of 198 accessions. Chromosome numbers are based on *M. truncatula*

Treat-ment	Trait	Marker	Chr	P value	R^2^	Associated with other traits	Annotated gene
CTL	ADF	S1_110050752	4	5.86E-05	0.08	DDM,ENE,IVTDMD30,IVTDMD48,ME,NEG,NEL,NEM,CP,TDN	
CTL	DMI1	S1_305729816	7	5.68E-05	0.11	IVTDMD30,IVTDMD48,NDFD48,CP, RFQ,TDNL	programmed cell death protein
CTL	ADF	S1_231443201	7	9.34E-05	0.08	DDM,ENE,ME,NEG,NEL,NEM,TDN,TDNL	
CTL	ASH	S1_276968305	U	5.83E-07	0.15	FAT,IVTDMD48,NDFD48,TDNL	
CTL	IVTDMD48	S1_351118210	6	7.44E-05	0.11	NDFD48,NFC,RFQ,TDNL	
CTL	dNDF30	S1_157382372	8	8.21E-05	0.11	Lignin,NFC	
CTL	DMI1	S1_176594850	5	9.97E-05	0.10	DMI,FAT	hypothetical protein
CTL	ASH	S1_220027052	7	3.15E-06	0.12	NFC	
CTL	ASH	S1_371197359	2	4.46E-06	0.15	TDNL	reticulon-like protein B2
CTL	ASH	S1_7257201	1	9.57E-05	0.08	TDNL	
CTL	FAT	S1_301932961	U	8.36E-05	0.10	TDNL	
CTL	NFC	S1_201458525	6	1.67E-05	0.10	TDNL	
CTL	NFC	S1_153379823	4	4.19E-06	0.15		cadmium/zinc-transporting ATPase
CTL	ASH	S1_121767806	4	6.37E-05	0.09		
CTL	ASH	S1_271419513	8	7.47E-06	0.11		
CTL	ASH	S1_280254383	U	9.57E-05	0.10		
CTL	ASH	S1_292633486	U	2.14E-05	0.11		
CTL	ASH	S1_371197348	U	5.12E-05	0.13		
CTL	ASH	S1_60623914	2	2.29E-05	0.09		
CTL	dNDF30	S1_322005811	U	1.15E-05	0.10		
CTL	IVTDMD30	S1_62489807	2	6.63E-05	0.09		
CTL	IVTDMD48	S1_66277800	3	9.85E-05	0.12		
CTL	Lignin	S1_333201923	U	7.53E-05	0.11		
CTL	NDFD48	S1_212677714	7	9.72E-05	0.08		
CTL	NDFD48	S1_250094545	8	6.34E-05	0.12		
CTL	NFC	S1_314670933	U	3.91E-05	0.11		
CTL	CP	S1_164595549	7	8.47E-06	0.10		
CTL	CP	S1_247656456	8	4.15E-05	0.12		
CTL	CP	S1_258595951	8	8.41E-05	0.10		
CTL	CP	S1_28009323	1	6.19E-05	0.10		
CTL	CP	S1_385819739	U	7.88E-05	0.08		
CTL	RUP	S1_110050740	7	4.92E-06	0.11		
CTL	RUP	S1_185177363	5	2.30E-05	0.12		
CTL	RUP	S1_217816246	7	4.70E-05	0.10		
CTL	TDNL	S1_162648265	5	1.22E-05	0.10		
CTL	TDNL	S1_316254019	U	1.39E-05	0.11		
CTL	TDNL	S1_373469214	U	4.59E-05	0.09		
CTL	TDNL	S1_378651194	U	1.56E-05	0.12		
CTL	TDNL	S1_63800922	3	6.22E-06	0.14		
CTL	TDNL	S1_75158591	3	4.51E-05	0.09		
Mild	ASH	S1_292679040	1	2.49E-13	0.27	dNDF48,NDFD48, NFC,TDNL	cytochrome P450 family protein
Mild	ASH	S1_197238737	6	5.79E-16	0.38	dNDF48,NDFD48,NFC,RUP,TDNL	
Mild	ASH	S1_136859347	4	4.05E-14	0.31	NDFD48,NFC,TDNL	
Mild	ASH	S1_300540244	7	2.14E-15	0.38	NDFD48,NFC,TDNL	UDP-glucosyl-transferase family protein
Mild	ASH	S1_311409163	U	1.53E-10	0.21	NDFD48,NFC,TDNL	exostosin family protein
Mild	ASH	S1_45016191	2	4.05E-07	0.16	NDFD48,NFC,TDNL	
Mild	ASH	S1_62932631	2	1.20E-14	0.32	NDFD48,NFC,TDNL	helix loop helix DNA-binding domain protein
Mild	ASH	S1_62932652	2	1.20E-14	0.32	NDFD48,NFC,TDNL	
Mild	dNDF30	S1_50692984	2	4.72E-07	0.16	dNDF48,ASH,NFC	
Mild	ASH	S1_151845055	5	5.68E-09	0.21	NFC,TDNL	
Mild	ASH	S1_163809114	5	1.15E-06	0.17	NFC,TDNL	
Mild	ASH	S1_177465248	5	2.27E-05	0.12	NFC,TDNL	
Mild	ASH	S1_220027044	7	8.39E-06	0.15	NFC,TDNL	
Mild	ASH	S1_309228882	8	5.33E-08	0.18	NFC,TDNL	carotenoid cleavage dioxygenase
Mild	aNDF	S1_366420904	U	7.21E-05	0.09	IVTDMD30, IVTDMD48	
Mild	ASH	S1_130145842	4	3.78E-07	0.17	NFC,TDNL	RING zinc finger protein
Mild	ASH	S1_163809083	5	1.10E-05	0.14	NFC,TDNL	
Mild	ASH	S1_294827153	2	5.09E-09	0.23	NFC,TDNL	auxin-binding protein ABP19a
Mild	ASH	S1_387336049	3	3.82E-07	0.16	NFC,TDNL	
Mild	ASH	S1_96848449	3	1.82E-06	0.16	NFC,TDNL	
Mild	NFC	S1_380790483	U	1.75E-05	0.13	NFC,TDNL	
Mild	dNDF30	S1_11398797	3	1.11E-06	0.16	dNDF48	
Mild	dNDF30	S1_121767803	6	5.76E-07	0.17	dNDF48	
Mild	dNDF30	S1_140803955	4	6.87E-07	0.16	dNDF48	prolyl 4-hydroxylase subunit alpha-like protein
Mild	dNDF30	S1_20593175	1	6.68E-07	0.16	dNDF48	LRR receptor-like kinase
Mild	dNDF30	S1_255810503	8	6.88E-07	0.16	dNDF48	
Mild	dNDF30	S1_323917811	6	6.48E-07	0.16	dNDF48	
Mild	dNDF30	S1_324999216	5	1.04E-06	0.16	dNDF48	
Mild	dNDF30	S1_328564258	8	6.50E-07	0.16	dNDF48	
Mild	dNDF30	S1_352766048	7	7.37E-07	0.16	dNDF48	
Mild	dNDF30	S1_368369179	1	6.26E-07	0.16	dNDF48	HASTY 1
Mild	dNDF30	S1_41503160	2	8.40E-05	0.09	dNDF48	
Mild	dNDF30	S1_92116984	3	7.23E-08	0.16	dNDF48	OPT family oligopeptide transporter
Mild	dNDF30	S1_104541975	4	2.28E-07	0.17	dNDF48	
Mild	ASH	S1_217816276	7	5.75E-06	0.11	NFC	hypothetical protein
Mild	ASH	S1_244286074	8	3.92E-05	0.11	NFC	
Mild	ASH	S1_274178328	U	8.28E-08	0.19	NFC	E3 ubiquitin-protein ligase XBOS32
Mild	ASH	S1_97771859	3	1.66E-05	0.11	NFC	
Mild	ASH	S1_146032006	4	1.34E-05	0.11	TDNL	
Mild	IVTDMD48	S1_104542260	4	8.56E-05	0.09	TDNL	
Mild	IVTDMD48	S1_41574028	2	9.35E-05	0.12	NDFD48	
Mild	ASH	S1_109199212	4	9.23E-05	0.12		
Mild	ASH	S1_255810496	8	5.06E-06	0.14		
Mild	ASH	S1_274180547	U	1.97E-05	0.10		
Mild	ASH	S1_319319782	1	7.42E-06	0.11		IQ calmodulin-binding motif protein
Mild	ASH	S1_343058809	U	2.88E-05	0.13		
Mild	ASH	S1_344523780	U	6.40E-05	0.09		
Mild	ASH	S1_380790483	7	4.92E-08	0.19		feronia receptor-like kinase
Mild	ASH	S1_46291954	2	1.56E-05	0.15		
Mild	dNDF30	S1_73439383	3	9.68E-05	0.12		
Mild	FAT	S1_251411374	8	8.22E-05	0.09		
Mild	Lignin	S1_77578936	3	2.58E-05	0.10		
Mild	NFC	S1_4134311	1	4.89E-05	0.09		
Mild	NFC	S1_95240267	7	1.50E-06	0.15		
Severe	DMI1	S1_174013573	5	4.78E-05	0.10	DMI,FAT,RFQ,RFV	
Severe	ASH	S1_295104600	8	3.83E-12	0.25	NDFD48,NFC,TDNL	
Severe	DMI1	S1_223418326	7	6.33E-05	0.09	DMI,RFQ,RFV	
Severe	ASH	S1_3090630	1	2.17E-05	0.10	dNDF30,dNDF48	
Severe	ASH	S1_368127329	7	2.40E-07	0.16	NFC,TDNL	
Severe	ASH	S1_54810936	2	5.67E-06	0.13	NFC,TDNL	
Severe	NFC	S1_380541834	U	3.38E-05	0.12	ASH	
Severe	ASH	S1_108148173	4	4.90E-07	0.17	NFC	TIR-NBS-LRR family protein
Severe	ASH	S1_238285037	7	6.46E-05	0.10	NFC	
Severe	ASH	S1_259198355	8	5.03E-07	0.15	NFC	interferon-induced guanylate-binding protein
Severe	ASH	S1_261323300	8	3.13E-06	0.14	NFC	peroxidase family protein
Severe	ASH	S1_288329783	U	5.02E-06	0.13	NFC	
Severe	ASH	S1_303744644	U	4.95E-06	0.13	NFC	
Severe	ASH	S1_314656475	U	4.29E-06	0.13	NFC	
Severe	ASH	S1_351945756	U	1.06E-05	0.14	NFC	
Severe	ASH	S1_36657304	2	6.07E-06	0.13	NFC	
Severe	ASH	S1_86892057	3	4.10E-06	0.14	NFC	
Severe	ASH	S1_94485521	3	3.17E-06	0.14	NFC	
Severe	ASH	S1_42165100	2	4.07E-05	0.12	TDNL	
Severe	ASH	S1_138061496	4	5.62E-05	0.11		
Severe	ASH	S1_198430876	6	1.36E-06	0.12		
Severe	ASH	S1_20159111	1	1.27E-05	0.14		
Severe	ASH	S1_20221412	1	7.64E-06	0.13		
Severe	ASH	S1_208437908	7	3.99E-07	0.16		
Severe	ASH	S1_259969207	8	1.18E-05	0.14		
Severe	ASH	S1_314502177	U	4.76E-06	0.13		
Severe	ASH	S1_380541834	U	6.82E-06	0.14		
Severe	ASH	S1_62529190	2	3.89E-06	0.13		
Severe	ASH	S1_66246980	3	6.79E-05	0.09		
Severe	ASH	S1_97087353	3	1.64E-06	0.14		
Severe	FAT	S1_152463122	5	5.53E-07	0.16		
Severe	FAT	S1_213944479	7	9.20E-06	0.15		
Severe	FAT	S1_221806152	7	3.65E-05	0.10		
Severe	FAT	S1_260532035	8	4.56E-05	0.09		
Severe	FAT	S1_310998070	1	2.79E-07	0.15		RNA-binding (RRM/RBD/RNP motif) family protein
Severe	FAT	S1_323399172	U	8.83E-05	0.08		
Severe	FAT	S1_68331276	3	5.32E-07	0.15		
Severe	NFC	S1_313299937	U	1.99E-05	0.10		
Severe	RUP	S1_323002939	U	6.01E-05	0.09

### Identification of functional genes closer to the significant marker loci

Using the flanking sequences of the significant markers, we performed a BLAST search against the reference sequences to identify potential candidate genes underlying the significant marker loci. Of markers identified, 23 were found to be in close vicinity to known genes in the *M. truncatula* genome (Table 2). Among those identified under well-watered condition, marker S1_305729816 associated with DMI1 was in close vicinity to a programmed cell death. Marker S1_371197359 associated with ash was overlapped with reticulon-like protein B2. Marker S1_153379823 associated with NFC was adjacent to cadmium/zinc transporting ATPase. A number of markers were identified under mild water deficit, among them, marker S1_292679040 was adjacent to cytochrome P450 family protein; marker S1_300540244 was adjacent to UDP-glucosyltransferase; S1_311409163 was adjacent to exostosin; S1_62932631 was adjacent to helix loop helix DNA-binding domain protein; S1_309228882 was adjacent to carotenoid cleavage dioxygenase; S1_130145842 was adjacent to RING zinc finger protein; S1_294827153 was adjacent to auxin-binding protein ABP19a; S1_274178328 was adjacent to E3 ubiquitin protein ligase XBOS32; S1_319319782 was adjacent to IQ calmodulin-binding motif protein; and S1_380790483 was adjacent to Feronia receptor-like kinase. Three markers, S1_20593175, S1_368369179 and S1_92116984 associated with dNDF30 and dNDF48 were in close vicinity to LRR-receptor-like kinase, HASTY1 and OPT family oligopeptide transporter, respectively. Of those identified under severe water deficit, markers S1_108148173, S1_259198355 and S1_261323300 were adjacent to TIR-NBS-LRR resistance protein, interferon-induced guanylate-binding protein and peroxidase family protein, respectively. Another marker S1_310998070 associated with fat was adjacent to RNA-binding (RRM/RBD/RNP motif) protein.

## Discussion

### Mild drought intends to decrease fiber content and improve digestibility in alfalfa

Production of alfalfa for high quality requires an understanding of how environmental and forage management practices influence crop growth and development. Causations exists between the environment, plant response, and nutritive value. In general, yield and forage quality are inversely related. Under regular management, alfalfa yield increases most rapidly in early spring and summer, whereas the quality decreases during the hot summer. Cutting frequently for high-quality forage always induce low yield [19]. In previous study, we identified 22 markers associated with alfalfa yield under different water deficit conditions [20]. Of those, 15 markers were identical with the quality-related markers identified in this study, indicating that these loci might participate in controlling both the yield and quality traits in alfalfa. Interestingly, 14 of them are associated with dNDF30, implying the correlation between digestible neutral detergent fiber and yield under drought stress. It was reported that digestible NDF of forage can decrease over 40% in the maturity phase [21]. Lignification process is becoming active during maturity, lignin, cellulose and other complex carbohydrates are enriched and bound together to form vascular and xylem tissue, support plant growth and nutrient transportation system [22]. However, Lignin is an essentially indigestible compound. Lignin in the cell wall reduces the digestibility of cellulose and hemicellulose by rumen microbes [23]. Given that alfalfa quality is negatively correlated with yield during maturity, it is likely to suggest that the loci associated with NDF may also affect alfalfa yield under water deficit.

Any factor that retards plant development tends to promote the maintenance of forage quality. If a plant is stressed during growth, a shorter, finer-stemmed, leafier alfalfa is often produced. On the other hand, high temperature accelerates growth, tends to have a negative impact on forage quality. Alfalfa is relatively drought tolerant because its deeper root systems allow alfalfa to absorb deep soil water and quickly recover from drought conditions. However, when transpiration exceeds water absorption, a stress is imposed on the plant influencing metabolism, development, growth, and ultimately yield. Water deficit promotes a reduction in vegetative growth and promote early maturity. It has been suggested that mild drought stress may be beneficial for forage quality as drought-stressed alfalfa will accelerate its shift to reproductive growth [24]. Furthermore, the greater proportion of leaves in short-term of drought stress improve forage feed quality and digestibility [24]. However, if drought stress has been too severe, and for an extended period, plant stress is permanent and may not be recovered.

Alfalfa fiber is consisted of three components: cellulose, hemicellulose and lignin. Increasing fiber content of a forage generally decreases its energy content. Of the fiber fractions, cellulose is the major compound digested by the animal while lignin is virtually indigestible in both the rumen and lower intestines. In our study, drought decreased significantly both ADF, aNDF and lignin, which in turn increased energy-related traits such as TDN, ENE, DDM, NFC, RFV and RFQ. Cell wall remodeling is a common response of plants to abiotic stresses. Cellulose content in cell wall was significantly reduced as biomass composition drastically altered under drought stress. Drought stress increased cellulose conversion rates by enzymatic saccharification, affecting cell wall structural rigidity. Under drought stress, both cell wall composition and the extent of cell wall plasticity significantly variated among genotypes. However, only weak correlations were found between different levels of drought resistance, suggesting their independent genetic control.

### Genetic architecture of forage quality under well-watered and water deficit conditions

Among markers associated with forage quality under different irrigation episodes, a small number of the markers were in common between well-watered and water deficit conditions, while most of them responded dependently to the treatments (Fig. 6), suggesting their dependent genetic control. However, when phenotypic mean was used for GWAS, similar association patterns were found amongst energy-related traits, including DDM, TDN, ENE, ME, NEM, NEG and NEL, and traits of DMI, DMI1, RFV and RFQ. The genetic responses to mean values of these traits may suggest common genetic bases among them. This is logical as all these were energy-related traits.

In the GWAS, we only found nine associated markers that have consistent effects across water deficit treatments (Fig. 6b). The rests were differentially associated with respective treatments. Interestingly, about 2 folds of markers were associated with mild water deficit compared to those identified by severe water deficit (Fig. 6b), suggesting that mild water deficit affect genetic responses more favorably for forage quality traits than those in the severe stress and the control. In previous study, we identified 22 markers associated with biomass yield under water deficit in the same panel of accessions [20]. These markers were not found in the well-watered control. Similarly, in the present study, markers associated with forage quality under water deficit were not found in the control condition. In another study of genome-wide association and genetic selection on alfalfa quality traits, 10 SNP markers associated with acid detergent lignin (ADL), NDFD and CP were identified on chromosomes 1, 2, 4, 5, 7 and 8 in alfalfa [17]. Protein content is another important factor influencing alfalfa quality. In this study, we identified 14 markers associated with CP and RUP in all water conditions, indicating these markers may play common roles in protein-synthesis regardless water conditions. However, based on genetic positions, these markers were not overlapped with those reported previously [17]. This is probably due to that the genetic background of alfalfa germplasm and stress treatments used in two studies were different. Since drought tolerance is a complex trait and affected by genetic and environmental interaction (G x E), the allelic effect of associated causal variants may be influenced by the treatment of the stresses. Therefore, we cannot directly address whether conditionally neutral alleles accumulate genetic variation at a faster rate than constitutively expressed genetic variation. For example, the number of significant markers were significantly reduced when severe water deficit occurred compared to mild stress and well-watered control. This may indicate that the plants shut down some metabolic pathways to save energy to accomplish drought avoidance under severe drought stress. It was also possible that power of QTL detection was lower in severe drought conditions because of the lower variations.

### Putative candidate genes associated with forage quality

Among 23 annotated genes associated with forage quality traits, three genes were identified under well-watered condition (Table 2). The programmed cell death (PCD) protein was associated with DMI1, CP, RFQ, TDNL, NDFD48, IVTDMD-30 and IVTDMD-48. PCD in plants is a crucial component of development and defense mechanisms. It is an important process during the secondary cell wall formation in plants [25]. Its associations with multiple traits in the present study suggested that PCD involved in regulating forage quality in multiple ways. Reticulon-like protein B2 (RTNLB2) was associated with ash and TDNL. It has been reported that the RTNLB2 is located in endoplasmic reticulum and plays a role in regulating receptor transport to plasma membrane in Arabidopsis [26]. Another putative candidate, cadmium/zinc transporting ATPase (cadA) was associated with NFC. The cadA is located in vacuole and involved in cadmium and zinc or cobalt transport and may have a detoxification function through a vacuolar sequestration in Arabidopsis [27]. Fourteen genes were identified under mild water deficit (Table 2). Of those, cytochrome P450 was associated with ash, dNDF48, NDFD48, NCF and TDNL. P450 family protein is a large enzymatic protein family in plants and play a role in plant development and biotic and abiotic stresses responses [28]. Many P450 monooxygenases such as cinnamate 4-hydroxylase (C4H) and ferulate 5-hydroxylase (F5H) are key enzymes in lignin biosynthesis [29]. Sakiroglu et al. (2012) used F5H as one of the candidate genes in the lignin synthesis pathway and analyzed the haplotypes in exons 1 and 2 of F5H in three subspecies of diploid alfalfa and found that exon 2 had greatest number of SNPs. However, weak association was found between exon 2 of F5H and cell wall biosynthesis [30]. UDP-glucosyltransferase (UGT) was associated with ash, NDFD48, NFC and TDNL. UGT plays a role in abscisic acid (ABA) homeostasis which regulates the plant response to environmental stresses such as drought, cold and salinity [31]. In previous study, another gene encoding sugar transferase, identified on chromosome 8, is also related to several quality traits, such as ADF, NDF and xylose [9]. A RING zinc finger protein (RZFP) was associated with ash, NFC and TDNL under mild water deficit. It has been reported that overexpression of AtRZFP enhanced salt and osmotic tolerance through enhancing ROSs scavenging, maintaining Na^+^ and K^+^ homeostasis, adjusting the stomatal aperture to reduce water loss, and accumulating soluble sugars and proline to adjust the osmotic potential [32]. Proline and soluble sugar contents were increased when overexpression AtRZFP in Arabidopsis [32]. An E3 ubiquitin protein ligase XBOS32 was associated with ash and NFC. The role of E3 Ub-ligase in controlling protein turnover has been suggested by modifying UPS-related proteins and contributes to nuclear proteome plasticity during plant responses to environmental stress signals [33]. An IQ calmodulin-binding motif protein was associated with ash. Gao et al. [34] reported that a gene of osa-miR369c encoding IQ calmodulin-binding motif protein affected the regulation of plant growth under several abiotic stresses such as temperature, drought and salinity in rice. The identification of calmodulin-binding proteins in the present study supports the assumption that this regulator is important player in response to abiotic stress through the calcium-signaling pathway [35]. Five genes were identified under severe water deficit (Table 2). Among them, the TIR-NBS-LRR protein was associated with ash and NFC. The plant TIR-NBS-LRR gene family contains a large class of disease resistance genes [36]. The identification of the TIR-NBS-LRR gene associated with drought in present investigation suggested that this gene may play a role in drought response. There is evidence to suggest that overexpression of the NBS–LRR gene ADR1 enhanced drought tolerance in Arabidopsis and the ADR1 may play a role in signal transduction in a cross-talk in signaling network between disease resistance and drought tolerance [37]. An interferon-induced guanylate-binding protein (IIGBP) was associated with ash and NFC. The IIGBP is a GTPase induced by interferon and plays a role in directing inflammasome subtype-specific responses and their consequences for cell-autonomous immunity against a wide variety of microbial pathogens [38]. A peroxidase family protein was associated with ash and NFC. The peroxidase responses are directly involved in the protection of plant cells against adverse environmental conditions. Several roles have been attributed to plant peroxidases in response to biotic and abiotic stresses. A type III peroxidase RCI3 participated in the induction of HAK5, which is a high-affinity uptake transporter of potassium [39]. Moreover, peroxidases may have a cell wall cross-linking activity during plant defense mechanisms [40]. An RNA-binding (RRM/RBD/RNP motif) protein was associated with fat under severe water deficit. RNA-binding proteins (RBP) play important roles in post-transcriptional gene regulation. Recent investigation of plant RBPs demonstrated that, in addition to their role in diverse developmental processes, they are also involved in adaptation of plants to various environmental conditions [41]. Although the remaining genes identified under water deficit do not have direct roles in stress responses, they involve in diverse processes in cell developments. For instance, Auxin-binding protein (ABP) was associated with ash, NFC and TDNL under mild water deficit. It has been suggested that ABP1 in Arabidopsis is involved in a broad range of cellular responses to auxin, acting either as the main regulator of the response, such as interface for entry into cell division or, as a fine-tuning device as for the regulation of expression of early auxin response genes [42].

## Conclusion

In the present study, we evaluated 26 forage quality traits in a panel of 198 alfalfa accessions in the field trial under deficit irrigation gradience. Our results showed that water deficit decreased fiber contents and enhanced energy-related traits. The highest correlation coefficient was obtained between RFQ and the quality mean, supporting that the RFQ is more accurate in estimating overall forage quality compared to the RFV. Only a small number of markers were commonly associated with all treatments. Most of the associated markers were dependent on water deficit treatments, suggesting diverse genetic controls for forage quality traits in different levels of drought stress. Although GWAS on forage quality have been reported, we are the first to address the genetic base of phenotypic plasticity of forage quality traits under water deficits. The information gained from the present study will be useful for the genetic improvement of alfalfa by enhancing drought/salt tolerance while maintaining forage quality.

## Methods

### Plant materials

A panel of germplasm composited of 198 alfalfa accessions with potential drought tolerance were obtained from the USDA-ARS Western Regional Plant Introduction Station. Majority of the germplasms were collected in 1980s, in Canada and Northern US including British Columbia, Saskatchewan, Manitoba, Idaho, Montana, Nebraska, Washington, and North and South Dakota. The objective of the initial collecting project was to sample alfalfa stands that had survived 25 or more years in drought stressed environments. The remaining accessions were from different countries, including twelve collected from Afghanistan, two from China and Russia, and one from each of the following countries, Algeria, Bulgaria, India, Lebanon, Germany, Spain, Turkey, Oman and Yemen (Table S1). All plant accessions used in this study were provided by the USDA-ARS Western Regional Plant Introduction Station.

### Field experiments

Field experiments were conducted as previously described (Yu, 2017). Briefly, single representative plants of each of the 198 alfalfa accessions were clonally propagated. The cloned plants were transplanted to the field of the Roza Farm at the Irrigated Agriculture Research and Extension Center, Washington State University, Prosser WA, in 2016. Well-watered control, mild and severe drought stresses were applied to the plots as described (Yu, 2017).

### Alfalfa sampling and forage quality measurements

Shoot samples were collected from the field plots with each water stress treatment and they were subjected to quality analyses. Plant samples were dried in oven at 60 °C. They were then ground in Wiley Mill (Thomas Scientific, US) prior to the final grinding in Cyclotec 1093 sampling mill (Foss, Hillerød, Denmark) through a 1 mm screen. Sample powders were loaded and measured by Near Infrared Reflectance Spectroscopy (NIRS). Spectra were collected by a scanning monochromator (FOSS NIR Systems 6500, Silver Spring, MD, USA) in the spectral range from 400 to 2500 nm. A published NIRS Consortium equation 13AH50.2-eqa (NIRS Forage and Feed Testing Consortium, http://nirsconsortium.org) was used to predict quality factors.

### Statistical analysis

Phenotypic data were subjected to an analysis of variance with the random effect of genotype and the fixed effect of drought treatment. The regression plots (Fig. 1) and correlation coefficient (Fig. 3) were obtained using JMP13 (SAS Institute, NJ). Cluster analysis was performed using a nonlinear mapping method to investigate the relationships among 198 accessions using the combination of quality traits in the field experiments. Correlation analysis was done between the traits evaluated using the JMP Genomics (SAS Institute, NJ).

To estimate phenotypic plasticity, a plasticity index was calculated according to Valladares et al., [43] as follow:

$$ PI=\left({M}_{max}-{M}_{min}\right)/{M}_{max} $$

Where PI is plasticity index, M_max_ is the highest value of the treatment average and M_min_ is the lowest value of treatment average for a specific trait in the population.

### Genotyping by sequencing

Leaf samples were collected from individuals and used for DNA extraction using the Qiagen DNeasy 96 Plant kit, according to the manufacture’s protocol (Qiagen, CA). A methylation sensitive restriction enzyme, EcoT221 was used for DNA digestion, followed by library construction. Genomic libraries was sequenced using Illumina HiSeq2000. FastQC (v0.11.2) was used for initial quality check of the sequence reads (http://www.bioinformatics.babraham.ac.uk/projects/fastqc/). Process_Radtags built in Stacks was used for deconvoluting and cleaning sequencing reads [44]. The resulting reads with high quality were then aligned to the *M. truncatula* reference genome (Mt4.0 v1) (*www.phytozome.org/M**. truncatula*) using the Burrow Wheelers Alignment tool (Version 0.5.9) with default alignment parameters [45]. Loci with missing > 50%, MAF < 5% were removed. After filtering, 10,327 SNPs, with a mean individual depth of 27 X, were obtained. The Row data of GBS were submitted to the NCBI Sequence Read Archive with bioproject ID: PRJNA287263 and biosample accession numbers: AMN03779142 - SAMN03779330.

### Genome-wide association analysis

The filtered marker data were used for GWAS using TASSEL according to Bradbury et al. [46]. A mixed linear model was used for GWAS as previously described [20]. The Benjamini false discovery rate (FDR) of 0.05 was used as a threshold for identifying significant marker-trait association [47].

## Supplementary information

**Additional file 1: Table S1**. Alfalfa accessions used in GWAS for salt tolerance.

**Additional file 2.**

## Data Availability

All data generated or analyzed in this study are included in this article and the supplemental files. The raw GBS data were submitted to the NCBI database with the bioproject ID: PRJNA287263.

## Abbreviations

ABA: Abscisic acid
ADF: Acid Detergent Fiber
aNDF: Neutral Detergent Fiber analyzed with amylase
ANOVA: analysis of variance
CP: Crude Protein
CTL: Control
DM: Dry Matter
DDM: Digestible Dry Matter
DMI: Dry Matter Intake using NDF
DMI1: Dry Matter Intake using NDF and NDFD
ENE: Estimated Net Energy
FDR: False discovery rate
GBS: Genotyping by sequencing
GS: Genomic selection
GWAS: Genome-wide association studies
IVDDM30: 30-h In Vitro Digestible Dry Matter
IVDDM48: 48-h In Vitro Digestible Dry Matter
ME: Metabolizable Energy
NEM: Net Energy for Maintenance
NEG: Net Energy for Gain
dNDF30: 30-h Digestible NDF
dNDF48: 48-h Digestible NDF
NDFD48: 48-h NDFD
NEL: Net Energy for Lactation
NFC: Nonfibrous Carbohydrates
NIRS: Near-infrared spectroscopy
PCD: Programed cell death
PI: Plasticity index
QTL: Quantitative trait loci
RFQ: Relative Forage Quality Index
RFV: Relative Feed Value Index
RUP: Rumen Undegradable Protein
SNP: Single nucleotide polymorphism
TASSEL: Trait Analysis by aSSociation, Evolution and Linkage
TDN: Total Digestible Nutrients
TDNL: Total Digestible Nutrients for legume
